# Polymorphism study of nine SNPs associated with subjective response to alcohol in Chinese Han, Hui, Tibetan, Mongolian and Uygur populations

**DOI:** 10.1080/20961790.2018.1468538

**Published:** 2018-06-12

**Authors:** Qingtao Wei, Yi Ye, Fan Chen, Jienan Li, Hao Wu, Yingqiang Fu, Youyi Yan, Linchuan Liao

**Affiliations:** Department of Forensic Toxicological Analysis, West China School of Basic Medical Sciences and Forensic Medicine, Sichuan University, Chengdu, China

**Keywords:** Forensic science, forensic genetics, alcohol drinking, heavy drinking, subjective response, single nucleotide polymorphism, polymorphisms, genetic

## Abstract

Heavy alcohol drinking is a major public health problem, causing a large disease, social and economic burden in societies. Subjective response (SR) to alcohol is an intermediate characteristic of heavy drinking. A variety of candidate genes have been reported to be associated with SR to alcohol. In this study, we investigated nine single nucleotide polymorphisms (SNPs) related to SR to alcohol in healthy individuals from five Chinese ethnic groups, the Han, Hui, Tibetan, Mongolian and Uygur populations, and a total of 584 bloodstain samples were collected. The nine SNPs included four SNPs in alcohol-metabolizing genes (*ADH1B*, *ADH1C*, *ALDH2* and CYP2E1*5B) and five SNPs in genes of neurobiological pathways (*GABRA2*, *OPRM1*, *CHRNA3*, *HYKK* and *SLC6A4*). A SNaPshot analysis method was developed to type these SNPs simultaneously, and all samples were typed successfully. Statistical analyses of the allele frequencies indicated that the frequencies of all SNPs, except for *ADH1C*, showed varying degrees of difference in the five studied ethnic groups. Tibetans showed the highest frequencies of risk alleles for heavy drinking at most loci. The genetic polymorphic differences found in this study revealed the variation in genetic susceptibility to heavy drinking in the studied populations.

## Introduction

Alcohol is a psychoactive substance with dependence-producing properties that has been widely used in many cultures for centuries. The harmful use of alcohol is a major public health problem, causing a series of diseases including heart disease, stroke, cancers and liver cirrhosis [1,2], and social and economic burden in societies. Chronic and heavy alcohol drinking is related to an increased probability of alcohol use disorders [1], as well as many other diseases, such as liver disease and some kinds of cancers [2]. In addition to the complicated and multiple social–environmental factors, such as attitudes towards drinking and drunkenness, patterns of drinking, laws and regulatory frameworks and drinking culture [1], genetic factors also play an important role in the level of alcohol consumption. Previous studies report that genetic influences explain more than 50% of the risk for heavy drinking and operate through intermediate characteristics such as impulsivity and an individual's level of response to alcohol [3,4]. Low levels of response to alcohol, especially of the negative, sedating effects [3,5], lead to heavier drinking. In addition, some alcohol challenge studies found that individuals with greater stimulant effects after alcohol intake appear to drink more [6,7].

Genes associated with subjective response (SR) to alcohol can be approximately divided into two categories [1,8]. The first category comprises variants in genes involved in alcohol metabolism. Alcohol is first oxidized into acetaldehyde mainly by alcohol dehydrogenases (ADHs) and is then further oxidized into acetate by aldehyde dehydrogenases (ALDHs) [9]. Cytochrome P450 2E1 (CYP2E1) accounts for only a small part of the total alcohol metabolism, but it can be induced up to 10-fold after chronic alcohol consumption [10]. The efficiency of alcohol metabolism determines the sensitivity to alcohol-induced adverse reactions (flushing, headache, tachycardia, nausea, etc.). All three enzymes exhibit genetic polymorphisms [9,10] and ethnic variation [11,12]. *ADH1B*, *ADH1C*, *ALDH2* and CYP2E1*5B are the most extensively researched loci [13,14]. The second category comprises genetic polymorphisms involved in neurobiological pathways. Given the large number of neurotransmitter systems potentially involved in alcohol's subjective effects, numerous studies have focused on variants in opioidergic genes, GABAergic genes, serotonergic genes, etc. Among them, the GABAA α2 receptor subunit gene (*GABRA2*) [15,16], μ-opioid receptor gene (*OPRM1*) [17,18], serotonin transporter gene (*SLC6A4*) [19,20] and nicotinic acetylcholine receptors (*CHRNA5–CHRNA3–CHRNB4* gene cluster) [21] are found to be potentially associated with SR to alcohol.

Alcohol has a very strong presence in cultures of many minority ethnic groups in China. Chinese Hui, Uygur, Tibetan and Mongolian are the top 10 ethnic groups in China, with each group comprising over 5 million people (National Bureau of Statistics of the People's Republic of China) [22]. Epidemiological investigations indicate that Tibetan population (64.16% in male, 36.0% in female) [23] and Mongolian population (62.8% in male, 9.0% in female) [24] have a higher prevalence of drinking than Chinese residents (39.6% in male, 4.5% in female) [25]. In addition, the morbidity of alcohol use disorders in Tibetans [26] is much higher than in Chinese residents based on the data reported by the World Health Organization [27] and another survey [28]. Although the data of alcohol consumption of other ethnic groups are lacking, many anecdotal observations suggested that these minority ethnic groups drink more than the Chinese Han population [29].

In this study, the authors selected nine single nucleotide polymorphisms (SNPs) in the abovementioned genes, which were believed to be the most likely to affect the SR to alcohol based on previous studies, and developed a SNaPshot analysis method to type these SNPs simultaneously. The purpose of this study was to investigate the polymorphism distribution of these functional SNPs in the five mainland Chinese populations, Han, Hui, Tibetan, Mongolian and Uygur, and to explore the potential genetic differences in the SR to alcohol and the genetic susceptibility to heavy drinking in these populations.

## Material and methods

### Sample collection

In this study, a total of 584 samples were collected from unrelated mainland Chinese healthy individuals, including 122 Han, 112 Hui, 120 Tibetan, 112 Mongolian and 118 Uygur individuals. Each individual had the same ethnic origin for atleast three generations. Before sample collection, all the participants signed the informed consent after receiving an explanation about this study. Our study was approved by the Ethics Committee of the Sichuan University. The genomic DNA was extracted from bloodstain samples using the Chelex-100 method [30].

### SNP selection

According to previous studies [9,10,15–21], we focused on nine autosomal SNPs: four SNPs in alcohol-metabolizing genes and five SNPs in neurobiological pathways. The sequence variation and predicted phenotypes of these loci are presented in Table 1. Detailed sequences were obtained from GenBank (http://www.ncbi.nlm.nih.gov).

**Table 1.** Information about the SNPs studied in the present study.

Gene	SNP	Nucleotide change	Predicted phenotype of mutation
*ADH1B*	rs1229984	G > A	Higher oxidation activity [9]
*ADH1C*	rs698	G > A	Lower oxidation activity [9]
*ALDH2*	rs671	G > A	Almost inactivity [9]
CYP2E1*5B	rs2031920	C > T	Higher transcriptional activity [10]
*GABRA2*	rs279858	A > G	Lower stimulant and sedative effects [15]; greater stimulant effects [16]
*OPRM1*	rs1799971	A > G	Greater stimulant effects [17,18]
*CHRNA3*	rs1051730	G > A	Lower level of response [21]
*HYKK*	rs8034191	T > C	Lower level of response [21]
*SLC6A4*	rs1042173	T > G	Lower drinking intensity [19,20]

### SNaPshot technique

#### Primer design

Polymerase chain reaction (PCR) and extension primers were designed using Premier 6 software and Single Base Extension (SBE) Primer Version 1.1 software, respectively. To avoid overlap between the final SNaPshot products, a difference of 5–8 nucleotides between primer lengths was made by attaching 5′ non-homologous tails of poly (dTCCCC). The sequences of these primers are shown in Table 2.

**Table 2.** Primers used for SNaPshot.

Gene	SNP	Primer (5′→3′)
*ADH1B*	rs1229984	F- GGTCACCAGGTTGCCACTAAC
		R- TAGAAACACAATTTCAGGAATTTGGGTA
		Probe- GCCTCC(TCCCC)_1_CACTAACCACGTGGTCATCTGTG
*ADH1C*	rs698	F- TCCAGAGCGAAGCAGGTCAA
		R- ACTTGTGGCTGACTTTATGGCTAA
		Probe- GCCTCC(TCCCC)_7_(TC)AAGTTTTCACTGGATGCATTAATAACAAAT
*ALDH2*	rs671	F- GGAGCCCAGTCACCCTTTG
		R- TCCGAGCCACCAGCAGAC
		Probe- GCCTCC(TCCCCC)_3_ACGGGCTGCAGGCATACACT
CYP2E1*5B	rs2031920	F- GTGATTTGGCTGGATTGTAAATGACT
		R- GTGTGTGGTTAGAATGAAGAGAATGTT
		Probe- GCCTCC(TCCCC)_5_(TCCC)AAGTTCTTAATTCATAGGTTGCAATTTT
*GABRA2*	rs279858	F- TACAGCAGAGTCCCATCATCCT
		R- AGGTCCTATGAATATCCTTCGACTAA
		Probe- GCCTCC(TCCCC)_2_(TC)GGCATTGTCATATTATGAGCTACTGATTT
*OPRM1*	rs1799971	F-CGGTTCCTGGGTCAACTTGTC
		R-CACGCACACGATGGAGTAGAG
		Probe- GCCTCC(TCCCC)_4_(TCCC)TCAACTTGTCCCACTTAGATGGC
*CHRNA3*	rs1051730	F- GGATGATGAGGTTGATGGTGTAGAA
		R-TCTGGTCCTGATCGGCTCTTC
		Probe- CAGCAGTTGTACTTGATGTCGTGTTT
*HYKK*	rs8034191	F- ATTGGTCCTCTGATTGAGTAGTG
		R- CCCTGATTTCCACAAGTCCC
		Probe- GCCTCC(TCCCC)_5_(T) GCCCAATGTGGTATAAGTTTTCTGTT
*SLC6A4*	rs1042173	F- ACAGCAGCACATGGATTAGAAGG
		R- TGCGTAGGAGAGAACAGGGATG
		Probe- GCCTCC(TCCCC)_7_(TC) AAGGTTCTAGTAGATTCCAGCAATAAAATT

#### Multiplex PCR amplification and purification of PCR products

PCR reactions were carried out in a 5 μL final volume, including 2.5 μL of 2 × QIAGEN Multiplex PCR Master Mix (QIAGEN, Germany), 0.15 μL of each PCR primer (10 μmol/L) and 1 μL of template DNA. PCR conditions followed the product manuals of the QIAGEN Multiplex PCR Master Mix, with an annealing temperature of 60 °C. Multiplex amplification reactions were performed in a ProFlex™ 96-well PCR System (Thermo Fisher Scientific, USA). To remove excess primers and deoxy-ribonucleoside triphosphates (dNTPs), the PCR products were treated with 2.5 U of shrimp alkaline phosphatase (SAP) (TaKaRa Biotech, China) and 6 U of exonuclease I (TaKaRa Biotech, China) by incubation at 37 °C for 1 h, followed by enzyme inactivation at 80 °C for 10 min.

#### Multiplex SBE reactions and purification of SBE products

The extension reaction was performed in a 5 μL final volume containing 1.5 μL of PCR product, 0.15 μL of each extension primer (10 μmol/L) and 1.5 μL of SNaPshot reaction mix (Applied Biosystems, USA). The extension was performed for 25 cycles of denaturation at 96 °C for 10 s, annealing at 50 °C for 5 s and extension at 60 °C for 30 s. To remove unincorporated ddNTPs, the extension products were treated with 1 U of SAP by incubation at 37 °C for 1 h, followed by enzyme inactivation at 80 °C for 10 min.

#### Capillary electrophoresis

SBE products were separated by capillary electrophoresis on a 3130 DNA Genetic Analyser (Applied Biosystems, USA), and electrophoresis results were analysed using a GeneMapper® ID v.3.2 (Thermo Fisher Scientific, USA).

### Statistical analysis

Allele and genotype frequencies were computed using gene counting methods. The Hardy–Weinberg equilibrium analysis was carried out by Arlequin (version 3.5.1.2). Chi-squared test and Fisher's exact test (SPSS 20.0) were used to compare the genotype and allele distributions between different ethnic groups. *P* < 0.05 was considered to be significant in the comparisons.

## Results

**Figure 1.**
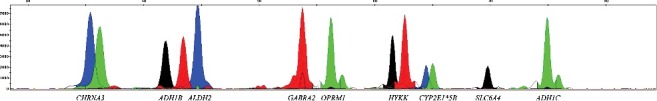
A typical electropherogram of one sample in the present study.

In this study, a SNaPshot multiplex assay was developed to type nine autosomal SNPs. All of the 584 bloodstain samples were typed successfully, and a representative electropherogram is shown in Figure 1. Except for CYP2E1*5B (rs2031920) in the Mongolian population (*P*-value of the Hardy–Weinberg equilibrium analysis, *P*_HWE_ = 0.018) and *SLC6A4* (rs1042173) in the Tibetan population (*P*_HWE_ = 0.046), all polymorphic sites in these populations were in Hardy–Weinberg equilibrium (Table 3).

**Table 3.** Genotype and allele frequencies of *ADH1B*, *ADH1C*, *ALDH2*, CYP2E1*5B, *GABRA2*, *OPRM1*, *CHRNA3*, *HYKK* and *SLC6A4* in Han (*n* = 122), Hui (*n* = 112), Tibetan (*n* = 120), Mongolian (*n* = 112) and Uygur (*n* = 118) populations.

		Han		Hui		Tibetan		Mongolian		Uygur	
Gene	Allele	Frequency (%)	*P*_HWE_	Frequency (%)	*P*_HWE_	Frequency (%)	*P*_HWE_	Frequency (%)	*P*_HWE_	Frequency (%)	*P*_HWE_
*ADH1B*	A/A	37.7	0.259	23.2	0.600	1.7	0.457	16.1	0.395	13.6	0.381
(rs1229984)	A/G	54.1		46.4		16.7		41.1		39.0	
	G/G	8.2		30.4		81.7		42.9		47.5	
		64.8		46.4		10.0		36.6		33.0	
		35.2		53.6		90.0		63.4		67.0	
*ADH1C*	A/A	70.5	0.334	83.9	1.000	71.7	1.000	76.8	1.000	78.0	0.581
(rs698)	A/G	29.5		16.1		26.7		21.4		20.3	
	G/G	0.0		0.0		1.7		1.8		1.7	
		85.2		92.0		85.0		87.5		88.1	
		14.8		8.0		15.0		12.5		11.9	
*ALDH2*	G/G	63.9	0.672	73.2	1.000	98.3	1.000	85.7	1.000	89.8	1.000
(rs671)	G/A	34.4		25.0		1.7		14.3		10.2	
	A/A	1.6		1.8		0.0		0.0		0.0	
		81.1		85.7		99.2		92.9		94.9	
		18.9		14.3		0.8		7.1		5.1	
CYP2E1*5B	C/C	62.3	0.459	58.9	0.424	88.3	1.000	66.1	0.018	69.5	1.000
(rs2031920)	C/T	31.2		39.3		11.7		23.2		28.8	
	T/T	6.6		1.8		0.0		10.7		1.7	
		77.9		78.6		94.2		77.7		83.9	
		22.1		21.4		5.8		22.3		16.1	
*GABRA2*	A/A	19.7	1.000	32.1	0.188	36.7	0.793	28.6	0.282	35.6	0.116
(rs279858)	A/G	49.2		41.1		46.7		57.1		39.0	
	G/G	31.2		26.8		16.7		14.3		25.4	
		44.3		52.7		60.0		57.1		55.1	
		55.7		47.3		40.0		42.9		44.9	
*OPRM1*	A/A	50.8	0.758	41.1	0.363	30.0	0.105	33.9	0.416	55.9	0.512
(rs1799971)	G/A	42.6		51.8		60.0		53.6		35.6	
	G/G	6.6		7.1		10.0		12.5		8.5	
		72.1		67.0		60.0		60.7		73.7	
		27.9		33.0		40.0		39.3		26.3	
*CHRNA3*	G/G	98.4	1.000	85.7	1.000	98.3	1.000	91.1	1.000	74.6	0.114
(rs1051730)	G/A	1.6		14.3		1.7		8.9		20.3	
	A/A	0.0		0.0		0.0		0.0		5.1	
		99.2		92.9		99.2		95.5		84.8	
		0.8		7.1		0.8		4.5		15.3	
*HYKK*	T/T	98.4	1.000	80.4	1.000	98.3	1.000	89.3	1.000	67.8	0.396
(rs8034191)	T/C	1.6		19.6		1.7		10.7		27.1	
	C/C	0.0		0.0		0.0		0.0		5.1	
		99.2		90.2		99.2		94.6		81.4	
		0.8		9.8		0.8		5.4		18.6	
*SLC6A4*	G/G	60.7	0.718	53.6	0.337	48.3	0.046	60.7	1.000	39.0	0.150
(rs1042173)	G/T	36.1		35.7		33.3		33.9		54.2	
	T/T	3.3		10.7		18.3		5.4		6.8	
		78.7		71.4		65.0		77.7		66.1	
		21.3		28.6		35.0		22.3		33.9	

*P*_HWE_: *P*-value of the Hardy–Weinberg equilibrium analysis.

The distribution of the *ADH1B* (rs1229984), *ADH1C* (rs698), *ALDH2* (rs671) and *CYP2E1* (rs2031920) genotypes and the frequencies of their respective alleles in the five ethnic groups are presented in Table 3. All five populations significantly differed from each other at the locus *ADH1B* with frequencies of the A-allele varying from 10.0% (in Tibetan) to 64.8% (in Han). No significant difference was found at *ADH1C*: all five populations had high frequencies of the A-allele (from 85.0% to 92.0%). Except for Hui, the other three populations showed significant differences compared with Han at *ALDH2*. The frequency of the A-allele in the Han population (18.9%) was higher than that of the Hui (14.3%), Mongolian (7.1%), Uygur (5.1%) and Tibetan (0.8%) populations. The Tibetans were significantly different from the other populations at CYP2E1*5B, with the lowest frequency of the T-allele (5.8%). The other four populations did not significantly differ from each other at this locus.

The allele and genotype distributions of *GABRA2* (rs279858), *OPRM1* (rs1799971), *CHRNA3* (rs1051730), *HYKK* (rs8034191) and *SLC6A4* (rs1042173) in the five populations are shown in Table 3. There were fewer differences among the populations at the loci of *GABRA2*, *OPRM1* and *SLC6A4*. The frequencies of the G-allele at *GABRA2* in Tibetans (40.0%) and Mongolians (42.9%) were statistically lower than Han (55.7%). Tibetans also had the highest frequencies of the G-allele at *OPRM1* and the T-allele at *SLC6A4*. The loci of *CHRNA3* (rs1051730) and *HYKK* (rs8034191) were in strong linkage disequilibrium, which was consistent with a previous study [21]. The frequencies of the minor alleles of both loci in Hui and Uygur were significantly higher than those of Han and Tibetans.

## Discussion

The PCR-restriction fragment length polymorphism method was common in SNP typing [31–33]. However, this method can be time-consuming and labour-intensive when multiple loci or large samples must be analysed. Thus, in the present study, we developed a SNaPshot multiplex assay, which was a medium-throughput method. Through its multiplexing capability, the nine studied SNPs could be analysed in a single reaction with high rates of accuracy and success.

Genetic variants in the genes of alcohol-metabolizing enzymes are generally associated with alcohol drinking, such as *ADH1B* and *ALDH2*. The hypothesized mechanism is that the isozymes encoded by these alleles lead to an accumulation of acetaldehyde, and excessive acetaldehyde accumulation causes intense adverse reactions leading to lower levels of alcohol intake [34]. Studies have confirmed that individuals carrying the ALDH2*A allele have higher levels of acetaldehyde [35] and more intense adverse reactions than ALDH2*G homozygotes and tend to have much lower levels of alcohol consumption [13]. However, the effects of *ADH1B* on drinking behavior are controversial; some studies indicate associations between ADH1B*A and facial flushing and alcohol consumption [14,36], but some studies are equivocal [37]. Similar frequencies were found between our data and previous polymorphism studies of ADH in Chinese ethnic groups [38,39]. The polymorphism distributions of *ADH1B* and *ALDH2* varied in Han, Hui, Tibetan, Mongolian and Uygur populations, suggesting that genetic sensitivity to acetaldehyde exposure, adverse reactions and alcohol consumption may differ in the diverse ethnic populations.

The low-level response theory proposed by Schuckit posits that less intense alcohol responses may be associated with insensitivity to internal cues and warning signs to stop drinking, resulting in excessive consumption [5]. The responses discussed in this theory are primarily sedative or negative intoxicating effects. However, heavy drinkers are found to experience greater stimulants and rewarding (liking and wanting) responses and lower sedative responses than light drinkers after alcohol consumption [6]. Both a low level of response and greater stimulant effects will lead to high levels of alcohol consumption. Our results indicated that the risk alleles, which were related to lower levels of response or greater stimulant effects, varied in the diverse populations.

One notable result is that the frequency distribution of Tibetans seemed to be very specific. Tibetans had very low frequencies of ADH1B*A and ALDH2*A, suggesting that Tibetans were less likely to suffer from alcohol-induced adverse reactions. Furthermore, the Tibetan population had the highest frequencies of risk alleles at *OPRM1* and *SLC6A4* when compared with the other populations. All the Tibetan samples in this study were collected from Lhasa, a high-altitude city in Tibet with extreme environments. In Tibet, alcohol use is not only culturally popular and encouraged but also physically adaptive. In addition, the genetic traits found in this study appeared to be a genetic permission for drinking. The social and genetic factors may partly explain the high prevalence of drinking and the high morbidity of alcohol-use disorders in Tibetans [23,26].

The genetic polymorphic differences found in this study revealed that the genetic susceptibility for heavy drinking varies in the diverse populations. This study may provide a framework for future studies regarding the genetic etiology of alcohol-related diseases in different ethnic groups.
